# Synthesis of Novel, Dual-Targeting ^68^Ga-NODAGA-LacN-E[c(RGDfK)]_2_ Glycopeptide as a PET Imaging Agent for Cancer Diagnosis

**DOI:** 10.3390/pharmaceutics13060796

**Published:** 2021-05-26

**Authors:** Barbara Gyuricza, Judit P. Szabó, Viktória Arató, Dániel Szücs, Adrienn Vágner, Dezső Szikra, Anikó Fekete

**Affiliations:** 1 Division of Nuclear Medicine and Translational Imaging, Department of Medical Imaging, Faculty of Medicine, University of Debrecen, Nagyerdei krt. 98, H-4032 Debrecen, Hungary; gyuricza.barbara@med.unideb.hu (B.G.); szabo.judit@med.unideb.hu (J.P.S.); arato.viktoria@med.unideb.hu (V.A.); szucs.daniel@science.unideb.hu (D.S.); szikra.dezso@med.unideb.hu (D.S.); 2 Doctoral School of Chemistry, Faculty of Science and Technology, University of Debrecen, Egyetem tér 1., H-4032 Debrecen, Hungary; 3 Doctoral School of Clinical Medicine, Faculty of Medicine, University of Debrecen, Nagyerdei krt. 98, H-4032 Debrecen, Hungary; 4 Doctoral School of Pharmaceutical Sciences, Faculty of Pharmacy, University of Debrecen, Nagyerdei krt. 98, H-4032 Debrecen, Hungary; 5 Scanomed Ltd., Nagyerdei krt. 98, H-4032 Debrecen, Hungary; vagner.adrienn@scanomed.hu

**Keywords:** radiopharmaceuticals, positron emission tomography (PET), α_v_β_3_ integrin, RGD peptide, gallium-68, radiochemistry

## Abstract

Radiolabeled peptides possessing an Arg-Gly-Asp (RGD) motif are widely used radiopharmaceuticals for PET imaging of tumor angiogenesis due to their high affinity and selectivity to α_v_β_3_ integrin. This receptor is overexpressed in tumor and tumor endothelial cells in the case of numerous cancer cell lines, therefore, it is an excellent biomarker for cancer diagnosis. The galectin-3 protein is also highly expressed in tumor cells and N-acetyllactosamine is a well-established ligand of this receptor. We have developed a synthetic method to prepare a lactosamine-containing radiotracer, namely ^68^Ga-NODAGA-LacN-E[c(RGDfK)]_2_, for cancer diagnosis. First, a lactosamine derivative with azido-propyl aglycone was synthetized. Then, NODAGA-NHS was attached to the amino group of this lactosamine derivative. The obtained compound was conjugated to an E[c(RGDfK)]_2_ peptide with a strain-promoted click reaction. We have accomplished the radiolabeling of the synthetized NODAGA-LacN-E[c(RGDfK)]_2_ precursor with a positron-emitting ^68^Ga isotope (radiochemical yield of >95%). The purification of the labeled compound with solid-phase extraction resulted in a radiochemical purity of >99%. Subsequently, the octanol–water partition coefficient (log *P*) of the labeled complex was determined to be −2.58. In addition, the in vitro stability of ^68^Ga-NODAGA-LacN-E[c(RGDfK)]_2_ was investigated and it was found that it was stable under the examined conditions.

## 1. Introduction

Positron emission tomography (PET) is a non-invasive, functional imaging technique that allows the visualization of physiological and pathological processes in the human body with a high sensitivity and selectivity. This imaging method requires a radiopharmaceutical that possess a positron emitter isotope attached to the targeting molecule. This vector molecule is responsible for the delivery of the radiation to a disease site. PET is capable of diagnosing diseases, monitoring disease progression, and detecting treatment response. The widespread clinical application of PET is for the visualization of glucose consumption of tumors and their metathesis using 2-deoxy-2-[^18^F]fluoro-D-glucose ([^18^F]FDG) as a radiopharmaceutical, which is a ^18^F-labeled analog of glucose.

Peptides can provide a more-specific cancer diagnosis compared to FDG-PET since they can bind to different tumor biomarkers with high affinity and specificity. Therefore, using peptides for the delivery of radionuclides to tumor cells is a promising strategy for diagnostic and therapeutic purposes [1]. Peptide-based radiopharmaceuticals are appropriate tools for patient selection who will respond positively to certain treatments.

The development of new blood vessels is essential for the transport of oxygen and nutrients for tumor cell growth [2]. During angiogenesis, new capillaries are created from pre-existing blood vessels; in healthy adults, this process rarely occurs and is occasionally triggered by hypoxia. Since tumor angiogenesis leads to tumor growth and malignant development, the inhibition of tumor neovascularization is an important approach to anti-cancer therapy [3]. Therefore, these receptors, which are overexpressed in the metastatic blood vessels, and not found (or are only present in a very small amounts) in normal blood vessels, are essential for this anti-cancer process. Among these biomarkers, the integrins, and especially the αvβ3 integrin, are revealed to be a valuable target for the detection and treatment of different cancer cells. The α_v_β_3_ integrin receptor is one of the key regulators of tumor growth, local invasiveness, metastatic potential, and the angiogenetic process [2,4]. Arg-Gly-Asp (RGD) peptide analogs are the effective ligands of α_v_β_3_ integrin and are widely used as biological vectors in different radiotracers, which are capable of detecting α_v_β_3_ integrin expression in tumor cells in preclinical and clinical investigations [4].

[^18^F]Galacto-RGD was first used for the imaging of α_v_β_3_ integrin expression in clinical studies [5]; however, its radiosynthesis is a complicated process and consists of four steps and three rounds of HPLC purification. The total synthesis time is about 200 ± 18 min and the radiochemical yield is 29.5 ± 5.1% (decay corrected) [6]. The radiolabeling of peptides via the complexation of radiometals with bifunctional chelators (BFCA) is a simpler and more effective procedure. The positron emitting ^68^Ga (t_1/2_ = 68 min, I = 89%, E_max_(β+) = 1.92 MeV) radiometal is a frequently used radionuclide for the labeling of peptides in preclinical and clinical studies, owing to the commercially available ^68^Ge/^68^Ga generator [7] and the successful clinical use of ^68^Ga-labeled somatostatin analogs for the imaging of neuroendocrine tumors [8].

Peptides usually show high selectivity for tumor cells, rapid tumor tissue penetration, and rapid clearance from non-target tissues and circulation. However, the moderate tumor uptake because of the short retention time frequently causes problem in the case of peptide probes, which contain small molecular weight peptides [9]. In addition to the applied vector molecule and chelator, the chemical structure of the linker unit also has a significant effect on the binding affinity, pharmacokinetics, and biodistribution of radiometal-based radiopharmaceuticals [10]. There are some chemical strategies to improve the pharmacokinetic performance of these radioactive agents, which are usually necessary for their clinical translation [9,10]. One of these is the glycosylation of peptides, which is a widespread method for improving tumor uptake and the pharmacokinetic profile of peptide-based radiotracers while enhancing their hydrophilicity and their penetration through biological membranes and reducing their metabolic degradation (e.g., [^18^F]Galacto-RGD [6]). For the glycosylation of the RGD peptide, we have selected a lactosamine derivative as a carbohydrate unit because *N*-acetyllactosamine is a well-established ligand of the galectin-3 protein [11]. Galectins are a family of lectins that recognize and bind to glycoconjugates and carbohydrates containing β-galactosides moieties through their carbohydrate-recognition domains. They are important modulators of different physiological and pathological processes, such as cell adhesion, proliferation, differentiation, as well as inflammation, angiogenesis, cancer progression, and metastasis [12]. Galectin-3 is highly expressed in different cancers [13], including melanoma [14] and colorectal cancer [15,16]. In addition, its higher expression correlates with a risk of metastases and poor survival; thus, galectin-3 was suggested as a prognostic marker [15,16]. Therefore, in addition to the above-mentioned advantages of peptides’ glycosylation, we expect that the incorporation of this carbohydrate unit into a precursor molecule might result in a dual-targeting radiopharmaceutical with a higher tumor uptake. Dual-targeted molecular imaging is also a promising method to enhance the binding performance of radioligands. Since the use of dual-targeting radiotracers can result in a higher tumor accumulation compared to a single-targeted probe due to the specific interaction with two different targets [17]. Successful application of the dual-targeting approach for improving imaging quality was reported in the case of peptide-based (e.g., ^18^F-labeled BBN-RGD [18], ^68^Ga-BBN-RGD [19,20]), antibody-based (e.g., ^64^Cu- and ^111^In-labeled trastuzumab fab-PEG24-EGF [21,22]), and nanoparticle-based (e.g., integrin α_v_β_3_/CD44-targeted nanoparticles [23]) imaging agents.

To prove this hypothesis, we have developed the synthesis and radiolabeling of a lactosamine-containing RGD derivative and investigated the in vitro stability of the labeled complex. To the best of our knowledge, this is the first report on the preparation of ^68^Ga-labeled E[c(RGDfK)]_2_-based glycopeptide, which possesses a lactosamine moiety for the detection of α_v_β_3_ integrin and galectin-3 expression in cancer and tumoral endothelial cells with PET imaging. The study of the in vivo kinetics and imaging properties of this novel radioligand is in progress.

## 2. Materials and Methods

### 2.1. General

All reagents and solvents were used without further purification. NODAGA-NHS chelating agent was purchased from Chematech (Dijon, France). cRGDfK dimer (E[c(RGDfK)]_2_) peptide was purchased from CASLO ApS (Lyngby, Denmark). All other reagents were purchased from Sigma-Aldrich. TLC was performed on Kieselgel 60 F_254_ (Merck, Kenilworth, NJ, USA) with detection by a UV detector and charring with 5% aqueous sulfuric acid. Column chromatography purification was performed on silica gel 60 (Merck 63–200 mesh). The ^1^H (400 MHz) and ^13^C NMR (128 MHz) spectra were recorded with Bruker DRX-400 spectrometer (Bruker, Billerica, MA, USA). Internal references were TMS (0.000 ppm for ^1^H) and CDCl_3_ (77.00 ppm for ^13^C for organic solution). Mass spectra were recorded on a Waters Acquity UPLC Iclass system (Waters, Milford, MA, USA) and a maXis II UHR ESI-QTOF MS Bruker instrument (Bruker, Billerica, MA, USA). For the HPLC system, HPLC-MS grade acetonitrile (ACN), MeOH (Fisher Solutions, El Cajon, CA, USA), and deionized water (Milli-Q, 18.2 MΩ cm^−1^, Merck, Kenilworth, NJ, USA) were used. ^68^Ga radioisotope was obtained from a GalliaPharm^® 68^Ge/^68^Ga isotope generator (Eckert-Ziegler Radiopharma, eluent: 0.1 M u.p. HCl, Berlin, Germany). Activity measurements were carried out with a CAPINTEC CRC-15PET dose calibrator and a Perkin Elmer Packard Cobra gamma counter (Llantrisant, UK). Semipreparative RP HPLC and analytical radio-HPLC were conducted using a Waters LC Module 1 HPLC and a Waters 2695 Alliance HPLC system, connected to a UV detector and the ATOMKI 120 CsI scintillation detector. Semipreparative RP HPLC was performed using a Luna C18 10 µm (250 × 10 mm) column; solvent A: 0.1% HCOOH; solvent B: ACN. Analytical HPLC was performed using a Luna C18 3 µm (150 × 4.6 mm) column, solvent A: oxalic acid (0.01 M, pH 3); solvent B: acetonitrile. Purification of labeled radiopharmaceuticals was carried out with the following chromatographic material: Oasis HLB SPE cartridge. Radio-TLC was performed on an iTLC-SG (Agilent Technologies, Santa Clara, CA, USA) and analyzed with a MiniGita TLC-Scanner using GINA-Star TLC software. Human plasma was obtained from Sigma-Aldrich (Saint Louis, MO, USA).

### 2.2. Chemical Synthesis

#### 2.2.1. 3-Azidopropyl 6-*O*-tert-butyldiphenylsilyl-2-phtalimide-2-deoxy-β-D-glucopyranoside (**2**)

The starting material, namely 3-azidopropyl 2-phtalimido-2-deoxy-β-D-glucopyranoside (**1**), was synthetized according to a previously described method [24]. To a solution of compound **1** (100 mg, 0.25 mmol) in dimethyl formamide (2 mL) were added t-BuPh_2_SiCl (80 µL, 0.31 mmol) and *N,N*-diisopropylethylamine (220 µL, 1.27 mmol). The reaction mixture was stirred overnight at room temperature, diluted with CH_2_Cl_2_, washed with aq. NaCl (5%), dried and then concentrated. The crude product was purified by column chromatography (Silica gel: 15 g, eluent: CH_2_Cl_2_-EtOAc 8:2, fractions: 20 mL) to yield **2** (132 mg, 82%) as a colorless syrup. ^1^H NMR (400 MHz, Chloroform-d) δ 7.88–7.79 (m, 2H), 7.76–7.66 (m, 6H), 7.50–7.32 (m, 6H), 5.20 (d, J = 8.4 Hz, 1H, H-1), 4.41–4.33 (m, 1H), 4.12 (dd, J = 11.0, 8.4 Hz, 1H), 3.96 (dd, J = 5.2, 2.6 Hz, 1H), 3.81 (ddd, J = 9.9, 6.1, 5.2 Hz, 1H), 3.69 (t, J = 8.9 Hz, 1H), 3.59 (dt, J = 9.8, 5.1 Hz, 1H), 3.47 (ddd, J = 9.9, 7.5, 5.0 Hz, 2H), 3.17–3.10 (m, 2H), 1.80–1.57 (m, 2H), 1.07 (s, 9H). (Supplementary Material Figure S1) ^13^C NMR (Chloroform-d): 135.74-127.80 (aromatic C), 98.26 (C-1), 74.50, 74.43 and 71.82 (C-3, C-4 and C-5), 66.04 (CH_2_) 65.26 (C-6), 56.36 (C-2), 48.15 and 28.96 (CH_2_), 26.91 (C(CH_3_)_3_), 19.32 (C(CH_3_)_3_). (Supplementary Material Figure S2) HRMS ESI calcd for: C_33_H_38_N_4_O_7_Si, 653.2402 [M+Na]^+^. Found: 653.2403 [M+Na]^+^. (Supplementary Material Figure S9)

#### 2.2.2. 3-Azidopropyl 2,3,4,6-tetra-*O*-acetyl-β-D-galactopyranosyl-(1→4)-6-*O*-tert-butyldiphenylsilyl-2-phtalimide-2-deoxy-β-D-glucopyranoside (**4**)

To a solution of 2,3,4,6-tetra-O-acetyl-β-D-galactopyranosyl trichloroacetimidate (**3**) (282 mg, 0.81 mmol) acceptor **2** (425.2 mg 0.675 mmol) and 4 Ǻ molecular sieves in dry CH_2_Cl_2_ (7 mL) was cooled to −50 °C in an argon atmosphere. After cooling, to the reaction mixture was added TMSOTf (55 µL, 0.30 mmol). The reaction mixture was stirred for 4 h at −50 °C. After stirring, the reaction mixture was diluted with CH_2_Cl_2_, washed with water, dried and concentrated. The crude product was purified by column chromatography (Silica gel: 70 g, eluent: CH_2_Cl_2_-EtOAc 9:1→8:2, fractions: 60 mL) to yield **4** (412 mg, 64%) as a colorless syrup. ^1^H NMR (500 MHz, Chloroform-d) δ 7.92–7.82 (m, 2H), 7.79–7.69 (m, 6H), 7.49–7.33 (m, 6H), 5.35 (d, J = 3.0 Hz, 1H), 5.26–5.18 (m, 2H), 4.97 (dd, J = 10.5, 3.4 Hz, 1H), 4.70 (d, J = 8.0 Hz, 1H), 4.48–4.42 (m, 1H), 4.17 (dd, J = 10.7, 8.6 Hz, 1H), 4.13–4.05 (m, 2H), 4.01–3.96 (m, 1H), 3.95–3.92 (m, 1H), 3.89–3.80 (m, 4H), 3.61–3.56 (m, 1H), 3.49 (ddd, J = 9.9, 7.3, 5.2 Hz, 1H), 3.18 (t, J = 6.7 Hz, 2H), 2.13 (s, 3H), 1.98 (s, 3H), 1.97 (s, 3H), 1.78–1.68 (m, 2H), 1.70 (s, 3H), 1.25 (d, J = 7.2 Hz, 1H), 1.11 (s, 9H). (Supplementary Material Figure S3) ^13^C NMR (Chloroform-d): 170.61, 170.26, 170.08 and 169.29 (CO), 136.07-127.80 (aromatic C), 101.36 and 97.98 (C-1 and C-1’), 81.28, 74.82, 71.34, 70.93, 69.62, 68.83 and 66.96 (C-2’, C-3, C-3’, C-4, C-4’, C-5 and C-5’), 65.31 (CH_2_) 62.02 and 61.41 (C-6, C-6’), 56.29 (C-2), 48.24 and 29.81 (CH_2_), 26.97 (C(CH_3_)_3_), 20.72, 20.65, 20.53 and 20.42 (CH_3_CO), 19.51 (C(CH_3_)_3_). (Supplementary Material Figure S4) HRMS ESI calcd for: C_47_H_56_N_4_O_16_Si, 983.3353 [M+Na]^+^. Found: 983.3358 [M+Na]^+^. (Supplementary Material Figure S10)

#### 2.2.3. 3-Azidopropyl β-D-galactopyranosyl-(1→4)-6-*O*-tert-butyldiphenylsilyl-2-amino-2-deoxy-β-D-glucopyranoside (**5**)

To a solution of compound **4** (100 mg, 0.1 mmol) in EtOH (5 mL) was added ethylenediamine (100 µL, 1.5 mmol). After 2 day of stirring at 65 °C, the reaction mixture was evaporated. The crude product was purified by column chromatography (Silica gel: 5 g, eluent: CH_2_Cl_2_-CH_3_OH 9:1→8:2, fractions: 5 mL) to yield **5** (44 mg, 63.8%) as a colorless syrup. ^1^H NMR (400 MHz, Methanol-d_4_) δ 7.82–7.78 (m, 4H), 7.54–7.25 (m, 6H), 4.57 (d, J = 7.7 Hz, 1H), 4.35–4.20 (m, 2H), 4.04–3.88 (m, 3H), 3.85–3.75 (m, 2H), 3.70 (dd, J = 11.5, 4.5 Hz, 1H), 3.65–3.56 (m, 2H), 3.55–3.42 (m, 6H), 3.34-3.32 (m, 1H), 2.74 (dd, J = 10.0, 8.0 Hz, 1H), 1.91 (t, J = 6.4 Hz, 2H), 1.07 (s, 9H). (Supplementary Material Figure S5) ^13^C NMR (Methanol-d_4_): 136.05–128.63 (aromatic C), 104.90 and 103.96 (C-1 and C-1’), 78.18, 77.20, 76.90, 75.58, 74.96, 72.53 and 70.29 (C-2’, C-3, C-3’, C-4, C-4’, C-5 and C-5’), 67.41 (CH_2_) 63.40 and 62.58 (C-6, C-6’), 58.16 (C-2), 49.85 and 30.23 (CH_2_), 27.39 (C(CH_3_)_3_). (Supplementary Material Figure S6) HRMS ESI calcd for: C_31_H_46_N_4_O_10_Si, 663.3062 [M+H]^+^ and 685.2881 [M+Na]^+^. Found: 663.3049 [M+H]^+^ and 685.2828 [M+Na]^+^. (Supplementary Material Figure S11)

#### 2.2.4. 3-Azidopropyl β-D-galactopyranosyl-(1→4)-2-amino-2-deoxy-β-D-glucopyranoside (**6**)

Compound **5** (3.1 mg, 0.0027 mmol) was dissolved in trifluoroacetic acid (1 mL). After stirring overnight at room temperature, the reaction mixture was evaporated. The residue was purified by size exclusion column chromatography (Sephadex LH-60) to yield **6** (14.6 mg, 98%). ^1^H NMR (400 MHz, Deuterium oxide) δ 4.78 (d, J = 7.8 Hz, 1H), 4.46 (d, J = 7.8 Hz, 1H), 4.07–3.95 (m, 2H), 3.92 (d, J = 3.4 Hz, 1H), 3.88–3.61 (m, 11H), 3.54 (dd, J = 10.0, 7.7 Hz, 1H), 3.45 (t, J = 6.6 Hz, 2H), 3.08 (dd, J = 10.6, 8.4 Hz, 1H), 1.93 (t, J = 6.5 Hz, 2H), 0.96 (d, J = 9.2 Hz, 2H). (Supplementary Material Figure S7) ^13^C NMR (Deuterium Oxide): 103.05 and 98.55 (C-1 and C-1’), 78.25, 75.41, 75.07, 72.45, 70.92, 70.73 and 68.49 (C-2’, C-3, C-3’, C-4, C-4’, C-5 and C-5’), 67.55 (CH_2_) 61.07 and 59.70 (C-6, C-6’), 55.46 (C-2), 47.91 and 28.13 (CH_2_). (Supplementary Material Figure S8) HRMS ESI calcd for: C_15_H_28_N_4_O_10_, 447.1703 [M+Na]^+^. Found: 447.1757 [M+Na]^+^. (Supplementary Material Figure S12)

#### 2.2.5. NODAGA-LacN (**8**)

To a solution of compound **6** (2.5 mg, 0.00589 mmol) in borate buffer (450 µL 0.1 M, pH 8.4) was added NODAGA-NHS chelator (**7**, 5.4 mg, 0.0073 mmol) in dry dimethylsulfoxide (100 µL). The reaction mixture was cooled 10 °C. After stirring overnight, the reaction mixture was concentrated. The crude product was purified by preparative RP-HPLC gave compound **8** (1 mg, 22%). For semipreparative RP-HPLC, a Luna C18(2) 100 Ǻ 10 µm (250 × 10 mm) column was eluted at flow rate 4 mL/min using the following solvents: solvent A: 0.1% HCOOH solvent, B: 95% acetonitrile, gradient: 0 min: 100% A, 2 min: 100% A, 32 min: 100% B, 40 min 100% B. The product was collected between 11.7 and 12.8 min. HRMS ESI cald for: C_30_H_51_N_7_O_17_, 782.3420 [M+H]^+^. Found: 782.3466 [M+H]^+^. (Supplementary Material Figure S13)

#### 2.2.6. DBCO-E[c(RGDfK)]_2_ (**11**)

To a solution of cRGDfK dimer peptide (E[c(RGDfK)]_2_ (**9**), 8 mg, 0.006 mmol) in DMSO (150 µL) were added DIPEA (10.5 µL, 0.06 mmol) and solution of DBCO-NHS ester (**10**, 5 mg, 0.012 mmol) in DMSO (150 µL). The reaction mixture was stirred overnight at room temperature. Then the reaction mixture was concentrated. The crude product was purified by preparative RP-HPLC gave compound **11** (2.09 mg, 21.7%). The conditions of semipreparative RP-HPLC were the same as in the previous synthesis. The product was collected between 15.6 and 16.8 min. HRMS ESI calcd for: C_78_H_100_N_20_O_18_, 803.3840 [M+H]^2+^. Found: 803.3815 [M+H]^2+^. (Supplementary Material Figure S14)

#### 2.2.7. NODAGA-LacN-E[c(RGDfK)]_2_ (**12**)

The solution of compound **6** (2.5 mg, 0.00589 mmol) in borate buffer (450 µL, 0.1 M, pH 8.4) was cooled to 10 °C and then NODAGA-NHS chelator (7, 5.4 mg, 0.0073 mmol) in dry dimethylsulfoxide (100 µL) was added to it dropwise. The reaction mixture was left to reach room temperature. After stirring overnight, the reaction mixture was concentrated. The crude product was purified by preparative RP-HPLC and gave compound **12** (1 mg, 35%). The conditions of semipreparative HPLC were the same as the previous synthesis. The product was collected between 14.4 and 14.7 min. HRMS ESI calcd for: C_108_H_151_N_27_O_35_, 1216.0325 [M+2Na]^2+^. Found: 1216.0313 [M+2Na]^2+^. (Supplementary Material Figure S15)

### 2.3. Radiochemistry

#### 2.3.1. Investigation of ^68^Ga Labeling of NODAGA-LacN-E[c(RGDfK)]_2_ Using Different Ligand Concentrations (10, 17, 23 and 32 µM)

The ^68^Ge/^68^Ga-generator was eluted with 0.1 M aq. ultra-purified (u.p.) hydrochloric acid. The generator elute was fractioned by discarding the first 1.8 mL and collecting the next 1.2 mL for synthesis. A volume of 100 µL of ^68^GaCl_3_ eluate (approx. 6–8 MBq in 0.1 M HCl) was transferred into an Eppendorf vial, then 20 µL of NH_4_OAc buffer (3 M, pH 4) as well as 3, 5, 7 and 10 µL of aq. stock solution of NODAGA-LacN-E[c(RGDfK)]_2_ (1 mg/mL) were added to the ^68^GaCl_3_ solution, respectively. Thus, the applied ligand concentrations in the mixtures were as follows: 10, 17, 23 and 32 µM, respectively. The reaction mixtures were heated for 15 min at 95 °C. Then, the reaction mixtures were analyzed by radio-HPLC on Waters 2695 Alliance HPLC system with Luna C18 3 µm (150 × 4.60) column. Solvent A: 0.1% oxalic acid, solvent B: 95% acetonitrile, gradient: 0 min: 100% A, 1 min: 100% A, 10 min: 100% B, 11 min: 100% B, 12 min: 100% A.

#### 2.3.2. Investigation of ^68^Ga Labeling of NODAGA-LacN-E[c(RGDfK)]_2_ Using Different Temperatures (Room Temperature, 37, 60, 80 and 95 °C)

A volume of 100 µL of ^68^GaCl_3_ eluate (approx. 6–8 MBq in 0.1 M HCl) was transferred into an Eppendorf vial, then 20 µL of NH_4_Oac buffer (3 M, pH 4) as well as 10 µL of aq. Stock solution of NODAGA-LacN-E[c(RGDfK)]_2_ (1 mg/mL, 4.2 nmol) was added to the ^68^GaCl_3_ solution. The reaction mixtures were heated for 15 min at room temperature, 37, 60, 80 and 95 °C, respectively. Then the reaction mixtures were analyzed by the above-mentioned radio-HPLC method.

#### 2.3.3. Synthesis of ^68^Ga-NODAGA-LacN-E[c(RGDfK)]_2_ Radiotracer with Optimal Reaction Procedure

A volume of 1000 µL of ^68^GaCl_3_ eluate (approx. 60–80 MBq in 0.1 M HCl) was transferred into an Eppendorf vial, then 200 µL of NH_4_OAc (3 M, pH 4) as well as 100 µL of aq. stock solution of NODAGA-LacN-E[c(RGDfK)]_2_ (1 mg/mL, 42 nmol) was added to the ^68^Ga solution. The reaction was performed at 95 °C for 15 min. Then, the reaction mixture was passed through a pre-conditioned (5 mL EtOH, 10 mL water) SPE cartridge (Oasis HLB). After washing of the cartridge with 1 mL of water, the radiolabeled product was eluted with 1:1 mixture of ethanol and saline (2 × 250 µL). The purified radiolabeled complex was eluted with 50 vol% ethanol, concentrated and reconstituted with 100 µL saline. The radiochemical purity of the radiotracer was determined by the above-mentioned radio-HPLC method and was found to be >99%. In addition, the radiochemical purity was also determined by radio-TLC using iTLC and 0.5 M citrate buffer (pH 5.5) as an eluent which gave the same RCP.

#### 2.3.4. Determination of logP Value of ^68^Ga-NODAGA-LacN-E[c(RGDfK)]_2_

A volume of 50 µL of the purified ^68^Ga-NODAGA-LacN-E[c(RGDfK)]_2_ solution (approx. 2–3 MBq) was mixed with 450 µL of water and 500 µL of 1-octanol in an Eppendorf vial. The mixture was shaken with vortex shaker for 5 min and centrifugated (9000 rpm) for 5 min. An amount of 3 × 20 µL from the two solvents were pipetted into a vial. The radioactivity of the fractions was determined with a gamma counter.

#### 2.3.5. Determination of in Vitro Stability of 68Ga-NODAGA-LacN-E[c(RGDfK)]2 in Human Serum, Na_2_EDTA and Oxalic Acid

A total of 50 µL of the purified ^68^Ga-NODAGA-LacN-E[c(RGDfK)]_2_ solution (approx. 4–5 MBq) was added to 50–50 µL of human serum, Na_2_EDTA (0.01 M) and oxalic acid (0.01 M), respectively. The samples were analyzed by radio-HPLC at the beginning, as well as at 60 and 120 min. The analytical conditions were the same as those described before the quality control of the labeled compound.

## 3. Results and Discussion

### 3.1. Chemistry

For the design of the novel RGD-based radioligand, the following aspects were taken into consideration. The cRGDfK dimer peptide (**9**, E[c(RGDfK)]_2_) was chosen as vector molecule for tumor angiogenesis imaging, since the cyclic RGD analogs have shown a higher affinity to α_v_β_3_ integrin than linear peptides [25] and, in addition, the multimerization approach can also lead to enhanced tumor uptake [26]. We designed the application of 2-(4,7-bis(carboxymethyl)-1,4,7-triazonan-1-yl)pentanedioic acid (NODAGA) as a chelating agent; hence, 1,4,7 triazacyclononane-1,4,7 triacetic acid (NOTA) derivatives are capable of complexation of the ^68^Ga^3+^ ion with a high stability [27]. A lactosamine derivative was chosen as a carbohydrate unit for the glycosylation of E[c(RGDfK)]_2_, because *N*-acetyllactosamine is a natural ligand of galectin-3 [11]. This receptor is also highly expressed in different cancer cells [13]; therefore, the incorporation of this carbohydrate into the RGD based radiotracer can result in dual-targeting radiopharmaceuticals with improved targeting efficacies [17].

**Scheme 1:**
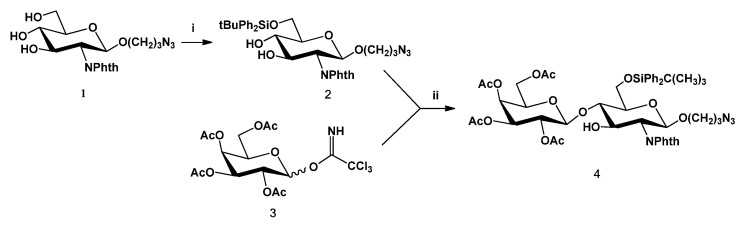
Reagents and conditions: (**i**) tBuPh_2_SiCl; DIPEA; DMF, rt, 24 h, 82%; (**ii**) TMSOTf; CH_2_Cl_2_, −50 °C, 4 h, 64%.

First, we developed a synthetic pathway for the preparation of the functionalized lactosamine derivative, which is suitable for conjugation to both E[c(RGDfK)]_2_ peptide (**9**) and NODAGA chelator. We designed the attachment of the lactosamine unit via azide-alkyne cycloaddition to the E[c(RGDfK)]_2_ peptide; therefore, we chose 3-azidopropyl 2-phtalimido-2-deoxy-β-D-glucopyranoside (**1**) [24] as the starting material. For the synthesis of the glycosyl acceptor **2**, the primary hydroxyl group of starting material **1** was silylated with tert-butyldiphenylsilyl chloride. Disaccharide **4** was prepared in the following way: glycosyl acceptor **2** was selectively glycosylated with the known 2,3,4,6-tetra-*O*-acetyl-β-D-galactopyranosyl trichloroacetimidate (**3**) [28] using trimethylsilyl trifluoromethanesulfonate as a catalyst in dichloromethane at −50 °C (Scheme 1).

**Scheme 2:**
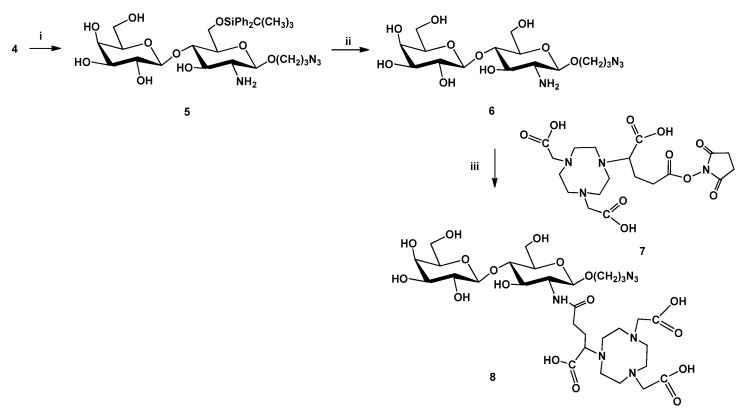
Reagents and conditions: (**i**) ethylenediamine, EtOH, 65°C, 48 h 64%; (**ii**) TFA, rt, 24 h, 98%; (**iii**) borate buffer (0.1 M, pH 8.4), DMSO, 10 °C→rt, 24 h, 22%.

Removal of the protecting groups from the disaccharide **4** was achieved in two steps. Firstly, the phtalimido and acetyl groups were removed with ethylene diamine in ethanol to yield compound **5**. Then the *tert*-butyl diphenylsilyl protecting group was removed with trifluoroacetic acid, which gave compound **6** (Scheme 2). In the next step, compound **6** was coupled with the commercially available NODAGA-NHS chelator (**7**) in dimethyl sulfoxide and in the presence of 0.1 M borate buffer (pH 8.4), which resulted in compound **8** (Scheme 2).

**Scheme 3:**
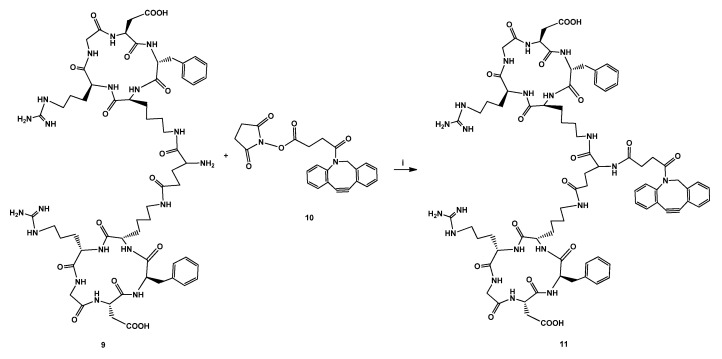
Reagents and conditions: (**i**) DIPEA, DMSO, rt, 24 h, 21.7%.

To avoid metal contamination, the attachment of the commercially available E[c(RGDfK)]_2_ peptide to chelator-bearing lactosamine **8** was designed with a copper-free, strain-promoted click reaction, which is a biorthogonal reaction of a type of azide-alkyne Huisgen cycloaddition. This method is widely used for the conjugation of biomolecules and was developed by Bertozzi et al. [29], which is based on the reaction of a cyclooctyne (e.g., dibenzocyclooctyne (DBCO)) moiety with an azide derivative and driven by the release of strain energy of the cyclooctyne ring. There are some examples of successful applications of this catalyst-free click reaction in the development of radiopharmaceuticals. Sapati et al. [30] used this conjugation method for the synthesis of [^64^Cu]DOTA-ADIBON3-Ala-PEG28-A20FMDV2 and found that the introduction of the chelator-strained alkyne (DBCO) system resulted in improved pharmacokinetics for their radiotracer. Jeon et al. [31] reported the radiolabeling of a DBCO-containing cRGD peptide and gold nanoparticle with ^125^I-labeled azide using a copper-free click reaction. They suggested this radiolabeling method for both in vitro and in vivo labeling of DBCO-containing molecules. Thus, the E[c(RGDfK)]_2_ peptide (**9**) was functionalized with DBCO moiety using commercially available DBCO-NHS (**10**) in dry dimethyl sulfoxide and in the presence of *N,N*-diisopropylethylamine (Scheme 3).

**Scheme 4:**
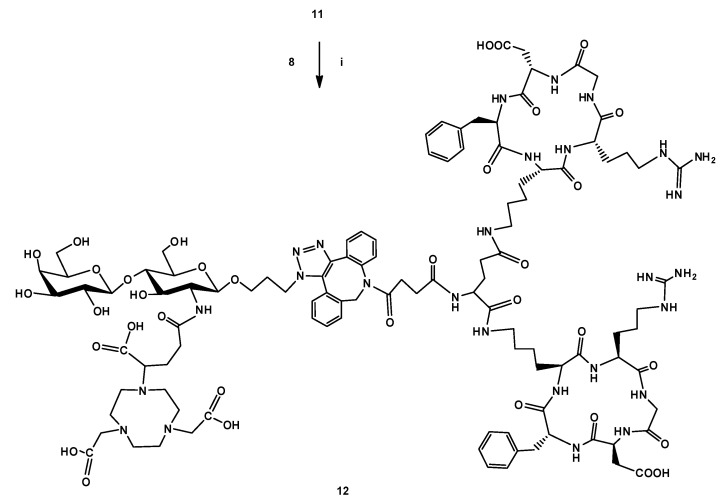
Reagents and conditions: (**i**) DMSO, rt, 24 h, 35%.

Finally, a strain-promoted click reaction was applied to the conjugation of DBCO-E[c(RGDfK)]_2_ derivative **11** to lactosamine derivative **8** in dimethyl sulfoxide (Scheme 4).

The synthetized NODAGA-LacN-E[c(RGDfK)]_2_ (**12**) was used for radiochemical investigations as a precursor molecule.

### 3.2. Radiochemistry

**Figure 1:**
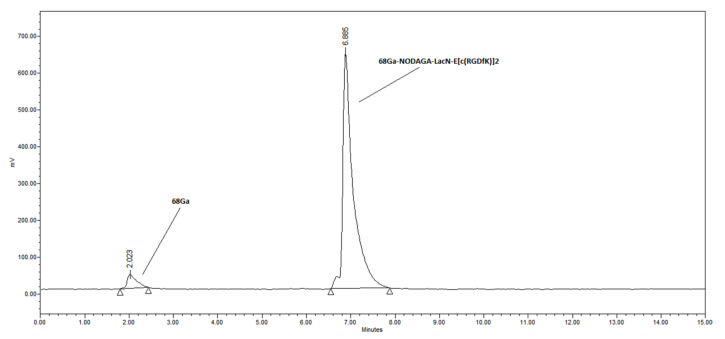
Radio-HPLC chromatogram of the reaction mixture using 32 µM ligand concentration and 15 min reaction time at 95 °C.

For the synthesis of ^68^Ga-NODAGA-LacN-E[c(RGDfK)]_2_, the ^68^Ga isotope was obtained from a ^68^Ge/^68^Ga-generator, which was eluted with 0.1 M ultra-purified (u.p.) HCl. Ammonium acetate buffer (pH 4, 3 M) and 1 µg/µL aqueous stock solution of NODAGA-LacN-E[c(RGDfK)]_2_ ligand were added to the ^68^GaCl_3_ solution. The labeling process was optimized regarding amount of peptide ligand and reaction temperature. The radiolabeling efficiency was characterized by determining the radiochemical purity (RCP) using radio-HPLC analysis of an aliquot from the crude reaction mixture. The radiolabelings were performed in triplicate for each ligand concentration and temperature (*n* = 3). For NODAGA-LacN-E[c(RGDfK)]_2_ radiolabeling, the highest radiochemical purity (~95%) was observed in the case of 32 µM ligand concentration for 15 min at 95 °C (Figure 1).

The application of the lower ligand concentrations resulted in slower kinetics, but in the case of the lowest ligand concentration (10 µM) the RCY was still ~85%. Details can be found in Table 1.

The change in temperature from room temperature to 95 °C resulted in a significant difference in the radiochemical yield. Thus, 95 °C was found to be the optimal temperature for the synthesis of ^68^Ga-NODAGA-LacN-E[c(RGDfK)]_2_ using a 32-µM ligand concentration and 15 min as a reaction time. The radiolabeling of the precursor with ^68^Ga isotope at 60 °C also resulted in an acceptable radiochemical yield (~92% RCP). However, no radiolabeling was observed at room temperature and the RCP was only ~8.53% at 37 °C. Details can be found in Table 2.

**Figure 2:**
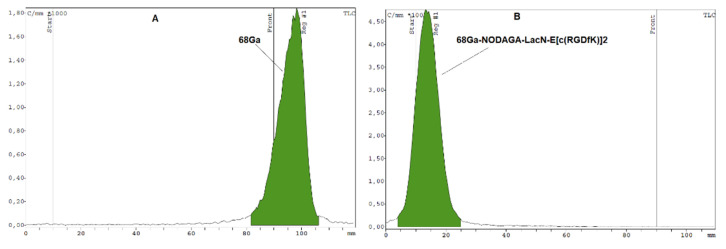
Radio-TLC chromatogram of free ^68^Ga^3+^ (**A**) and radio-TLC chromatogram of the purified radiotracer (**B**) using iTLC paper and 0.5 M citrate buffer (pH 5.5) as an eluent.

The following optimal labeling procedure was applied to further radiochemical experiments: 200 µL of NH_4_OAc buffer (3 M, pH 4) and 100 µL of aq. stock solution of NODAGA-LacN-E[c(RGDfK)]_2_ (1 µg/µL, 42 nmol) were added to 1000 µL of ^68^GaCl_3_ eluate (approx. 60–80 MBq in 0.1 M HCl). The reaction was conducted at 95 °C for 15 min. The reaction mixture was purified with solid phase extraction using a reversed phase Oasis HLB SPE cartridge. The radiochemical purity of the labeled complex was examined with radio-HPLC and was found to be more than 99%. In addition, we developed a radio-TLC method for quality control of the ^68^Ga-NODAGA-LacN-E[c(RGDfK)]_2_, applying iTLC paper and 0.5 M citrate buffer (pH 5.5) as an eluent, which gave the same RCP (Figure 2).

**Figure 3:**
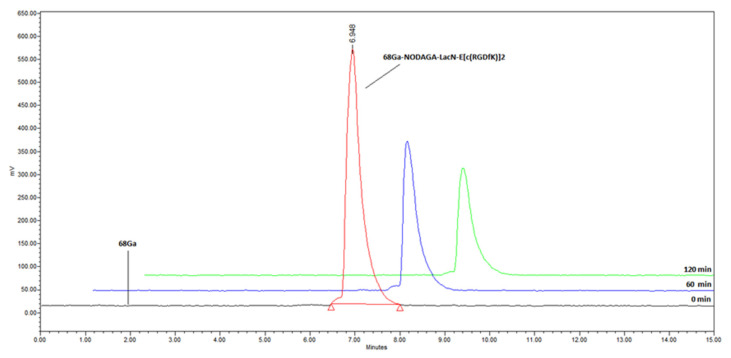
Human serum stability test.

The octanol/water partition coefficient (log*P*) of the ^68^Ga-NODAGA-LacN-E[c(RGDfK)]_2_ radioligand was determined and found to be −2.58. This low log*P* value indicated the hydrophilic nature of the synthetized radiotracer. To assess the stability, the labeled compound was incubated with a solution of human serum, Na_2_EDTA (0.01 M) and oxalic acid (0.01 M) at room temperature, respectively. Aliquots were then taken at different time points (0, 60 and 120 min) and injected into the radio-HPLC column and the chromatograms were analyzed. Figure 3 shows the results of the in vitro stability test of the radiotracer against human serum. According to the radio-HPLC chromatograms the ^68^Ga-NODAGA-LacN-E[c(RGDfK)]_2_ radiotracer remained intact (>99%) for two hours. The radio-HPLC chromatograms of the Na_2_EDTA and oxalic acid challenge showed the same result and the labeled compound remained stable for 2 h.

These stability studies proved that ^68^Ga-NODAGA-LacN-E[c(RGDfK)]_2_ possesses a high stability under the examined conditions over two hours.

## 4. Conclusions

In summary, we have developed a synthetic method for the preparation of a ^68^Ga-NODAGA-LacN-E[c(RGDfK)]_2_ radioligand containing lactosamine, which can be used for cancer diagnosis via PET imaging. NODAGA-NHS was attached to the amino group of a lactosamine derivative, which was functionalized with azido-propyl aglycone. Then, the obtained compound was conjugated with E[c(RGDfK)]_2_ peptide with copper-free, strain promoted click reaction. The radiolabeling of the synthetized NODAGA-LacN-E[c(RGDfK)]_2_ with a positron-emitting ^68^Ga isotope was carried out. After purification, the octanol–water partition coefficient of the labeled compound was determined and its stability was examined against human serum, Na_2_EDTA (0.01 M) and oxalic acid (0.01 M). We suppose that the synthetized, novel ^68^Ga-NODAGA-LacN-E[c(RGDfK)]_2_ radiopharmaceutical will be suitable for the detection of α_v_β_3_ integrin and galectin-3 expression in tumor and tumor endothelial cells with PET imaging. In further studies, we will assess the in vivo kinetics and imaging properties of this radiotracer.

## Figures and Tables

**Table 1:** Radiolabeling efficiency using different ligand concentration for 15 min at 95 °C (*n* = 3).

Ligand Concentration (µM)	Radiochemical Purity (%)
10	85.96 ± 5.02
17	90.80 ± 1.51
23	92.82 ± 1.13
32	95.02 ± 0.62

**Table 2:** Radiolabeling efficiency applying 32-µM ligand concentration and 15 min reaction time at different temperatures (*n* = 3).

Temperature (°C)	Radiochemical Purity (%)
room temperature	0
37	8.53 ± 1.06
60	92.84 ± 0.96
80	94.26 ± 0.72
95	95.02 ± 0.62

## Data Availability

Not applicable.

## Supplementary Materials

The following are available online at https://www.mdpi.com/article/10.3390/pharmaceutics13060796/s1, Figure S1: ^1^H NMR spectrum of compound 2, Figure S2: ^13^C NMR spectrum of compound 2, Figure S3: ^1^H NMR spectrum of compound 4, Figure S4: ^13^C NMR spectrum of compound 4, Figure S5: ^1^H NMR spectrum of compound 5, Figure S6: ^13^C NMR spectrum of compound 5, Figure S7: ^1^H NMR spectrum of compound 6, Figure S8: ^13^C NMR spectrum of compound 6, Figure S9: Mass spectrum of compound 2, Figure S10: Mass spectrum of compound 4, Figure S11: Mass spectrum of compound 5, Figure S12: Mass spectrum of compound 6, Figure S13: Mass spectrum of compound 8, Figure S14: Mass spectrum of compound 11, Figure S15: Mass spectrum of compound 12.
