# Supplementary material for: Synthesis of Novel, Dual-Targeting 68Ga-NODAGA-LacN-E[c(RGDfK)]2 Glycopeptide as a PET Imaging Agent for Cancer Diagnosis
Source: Pharmaceutics. 2021 May 26;13(6):796. doi: 10.3390/pharmaceutics13060796 (PMC8227980; doi:10.3390/pharmaceutics13060796)
Supplement: Supplementary file 1 [file pharmaceutics-13-00796-s001.zip › pharmaceutics-1224563-supplementary (1).pdf]

pharmaceutics

MDPI

Supplementary Materials: Synthesis of Novel, Dual-Targeting ^{68}Ga-NODAGA-LacN-E[c(RGDfK)]$_2$ Glycopeptide as a PET Imaging Agent for Cancer Diagnosis

Barbara Gyuricza, Judit P. Szabó, Viktória Arató, Dániel Szücs, Adrienn Vágner, Dezső Szikra and Anikó Fekete

Part 1: ^{1}H NMR and ^{13}C NMR spectra of compound 2, 4, 5 and 6

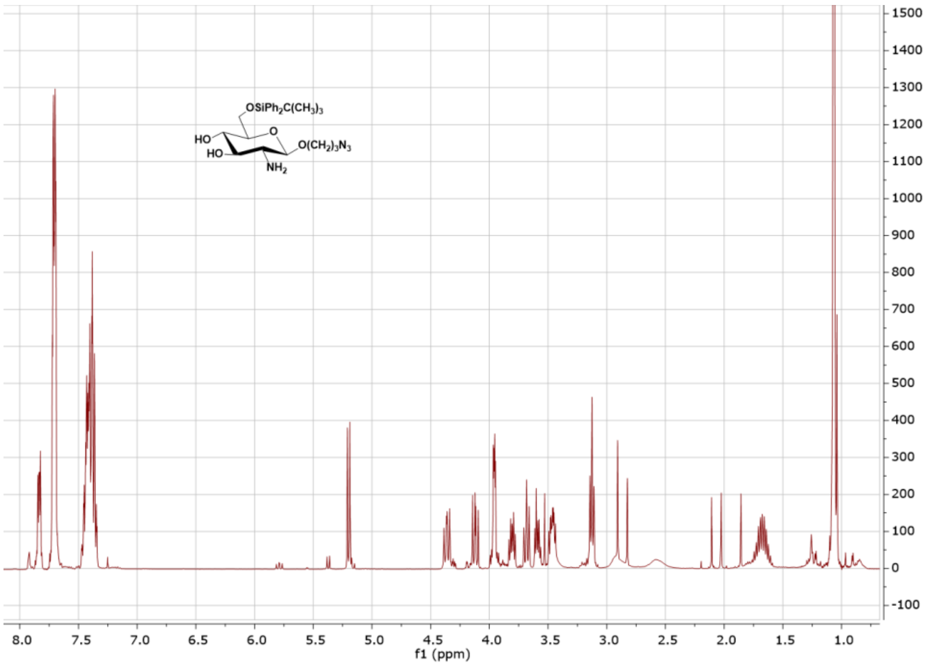

Figure S1. ^{1}H NMR spectrum of compound **2.**

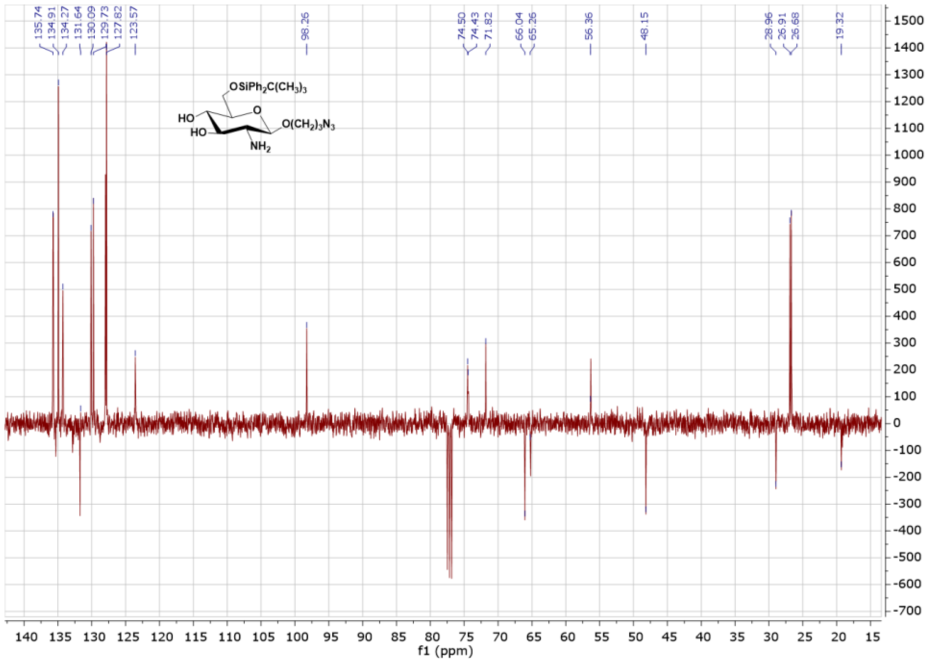

Figure S2. ^{13}C NMR spectrum of compound **2.**

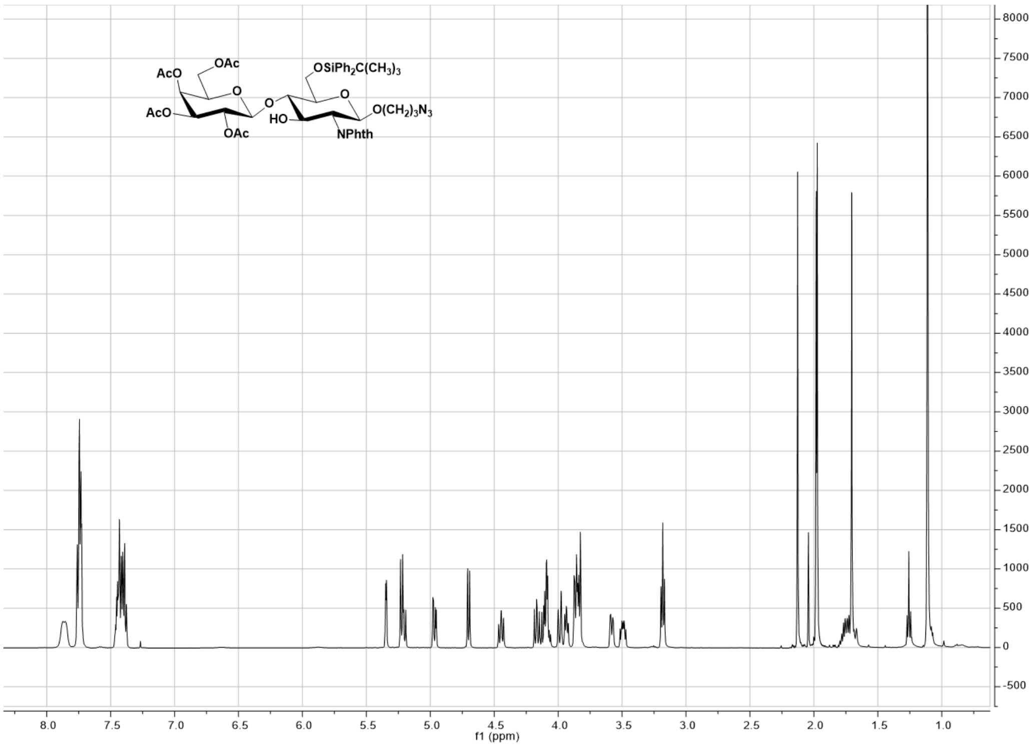

Figure S3. ^{1}H NMR spectrum of compound **4.**

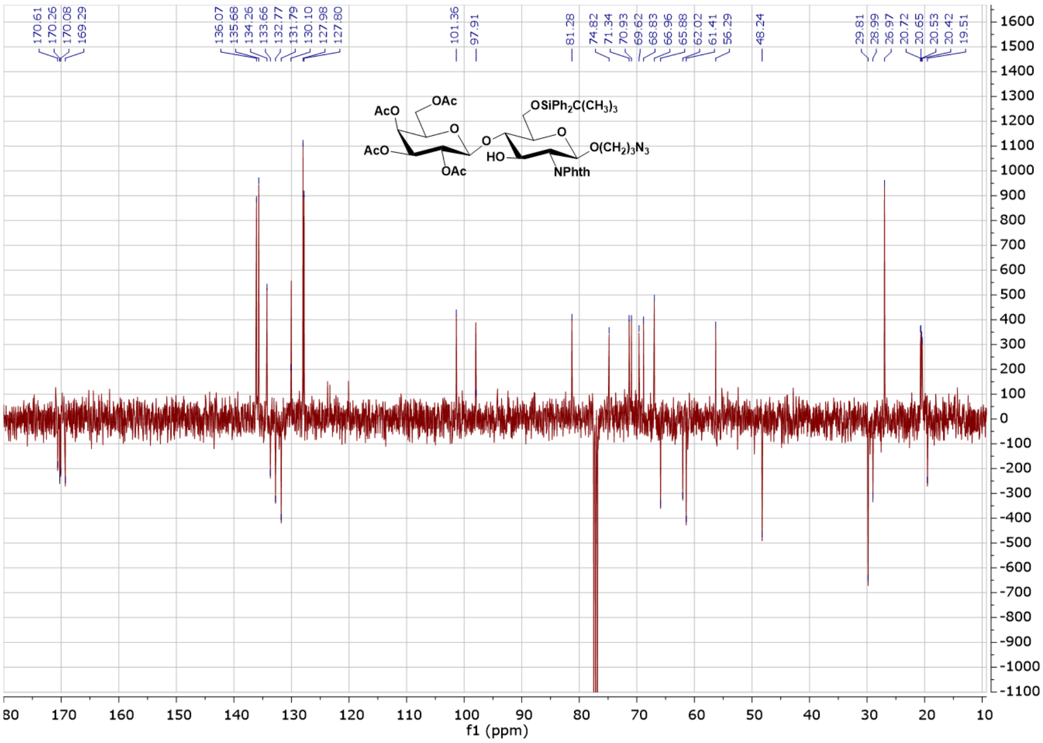

Figure S4. ^{13}C NMR spectrum of compound **4.**

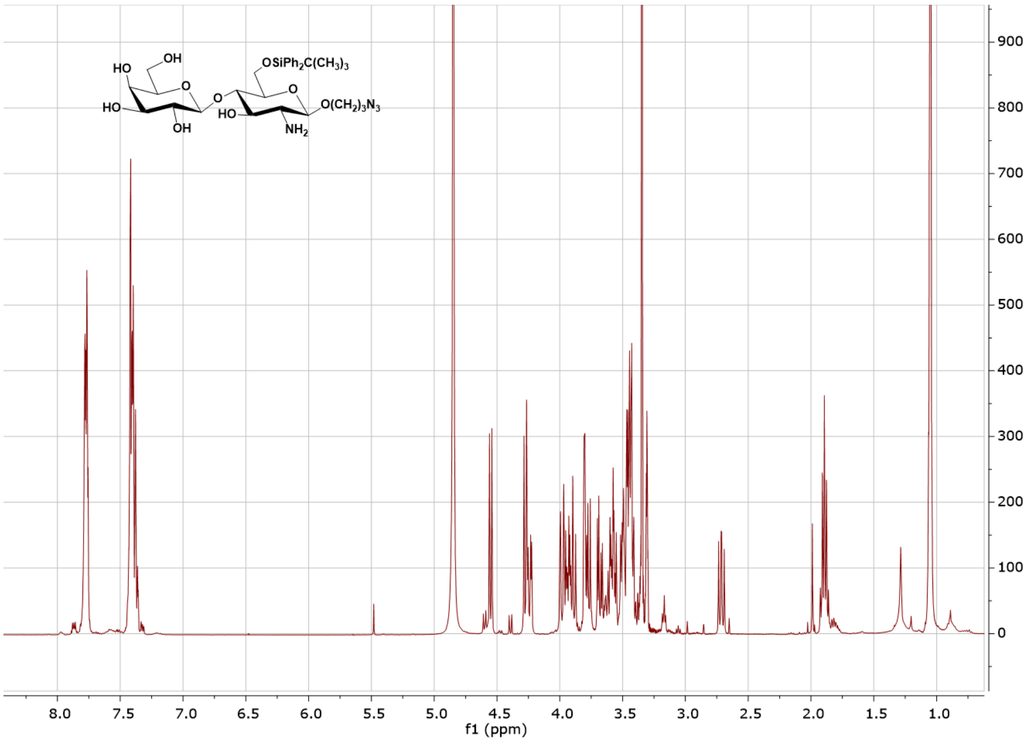

Figure S5. ^{1}H NMR spectrum of compound **5.**

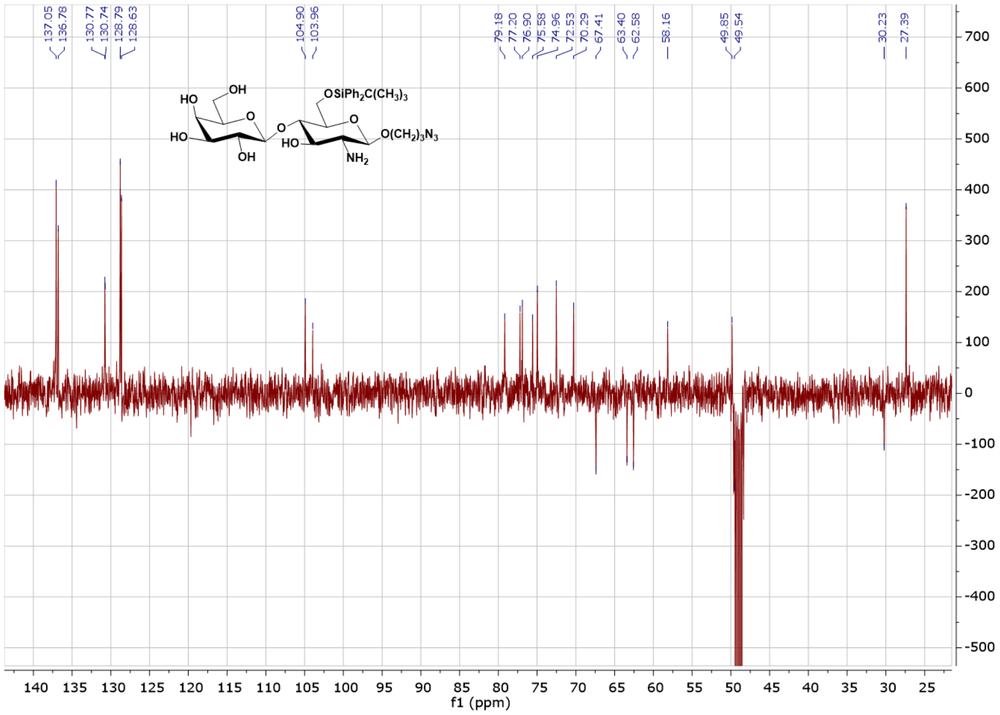

Figure S6. ^{13}C NMR spectrum of compound **5.**

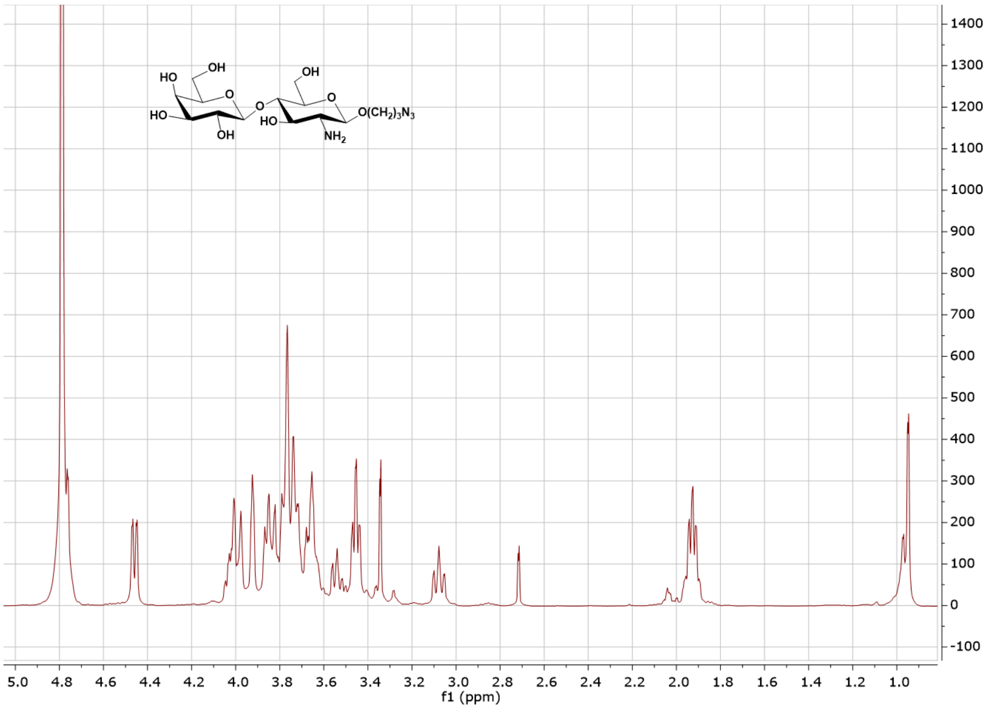

Figure S7. ^{1}H NMR spectrum of compound **6.**

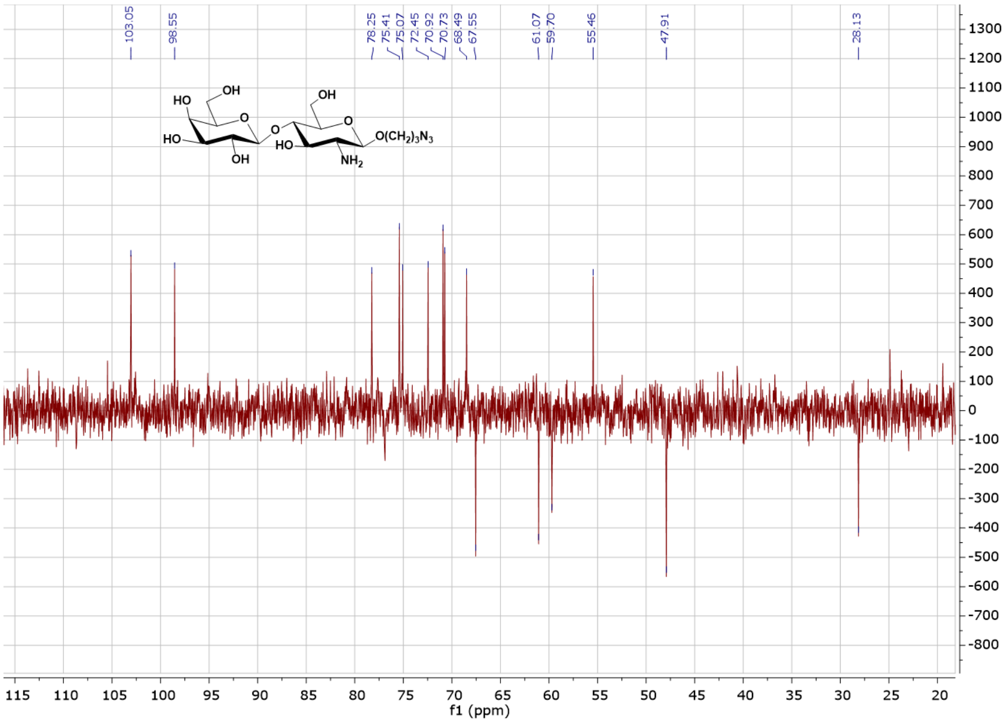

Figure S8. ^{13}C NMR spectrum of compound **6.**

Part 2: Mass spectra of compound 2, 4, 5, 6, 8, 11 and 12

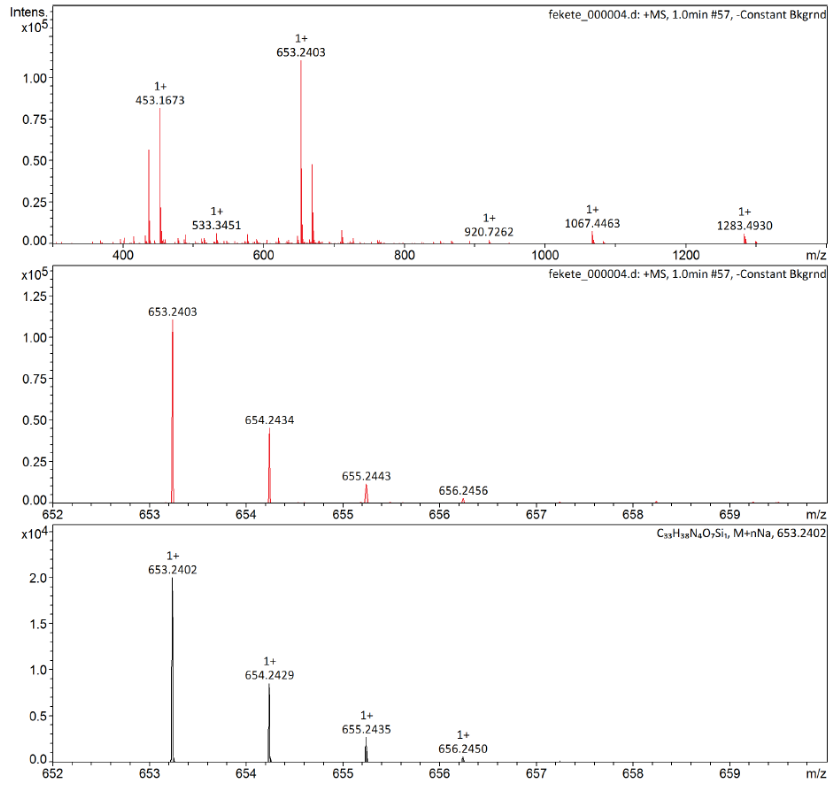

Figure S9. Mass spectrum of compound **2.**

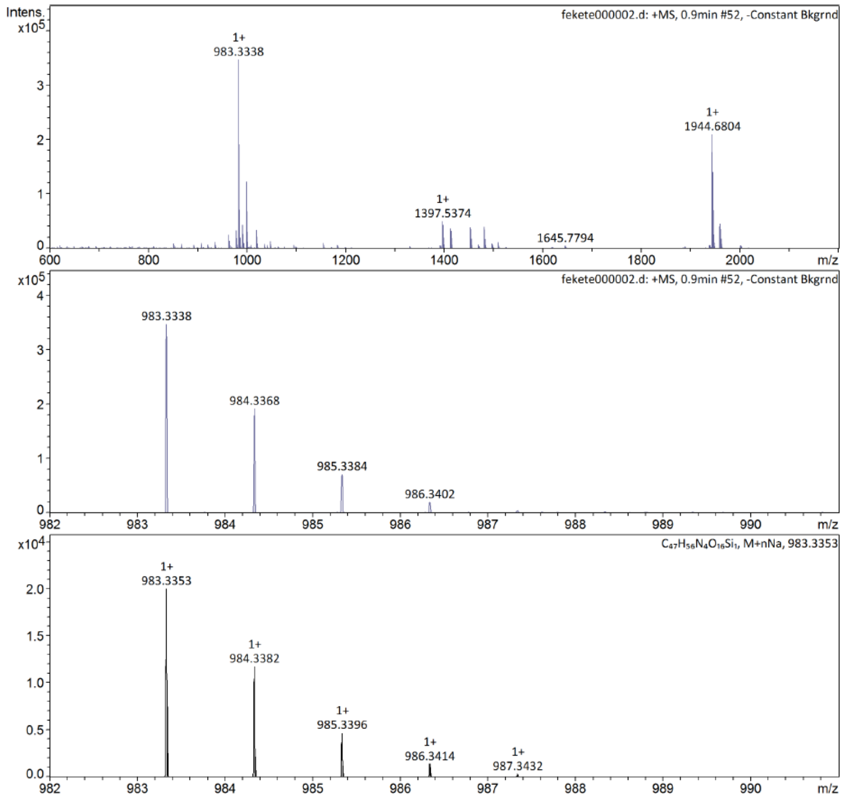

Figure S10. Mass spectrum of compound **4.**

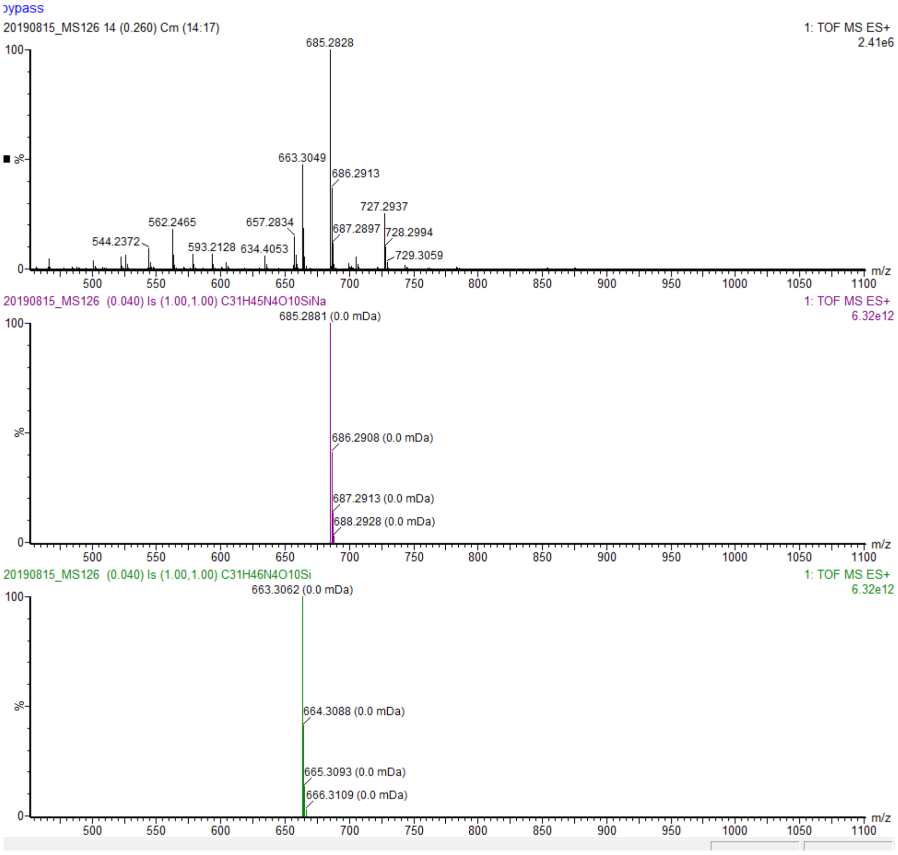

Figure S11. Mass spectrum of compound **5.**

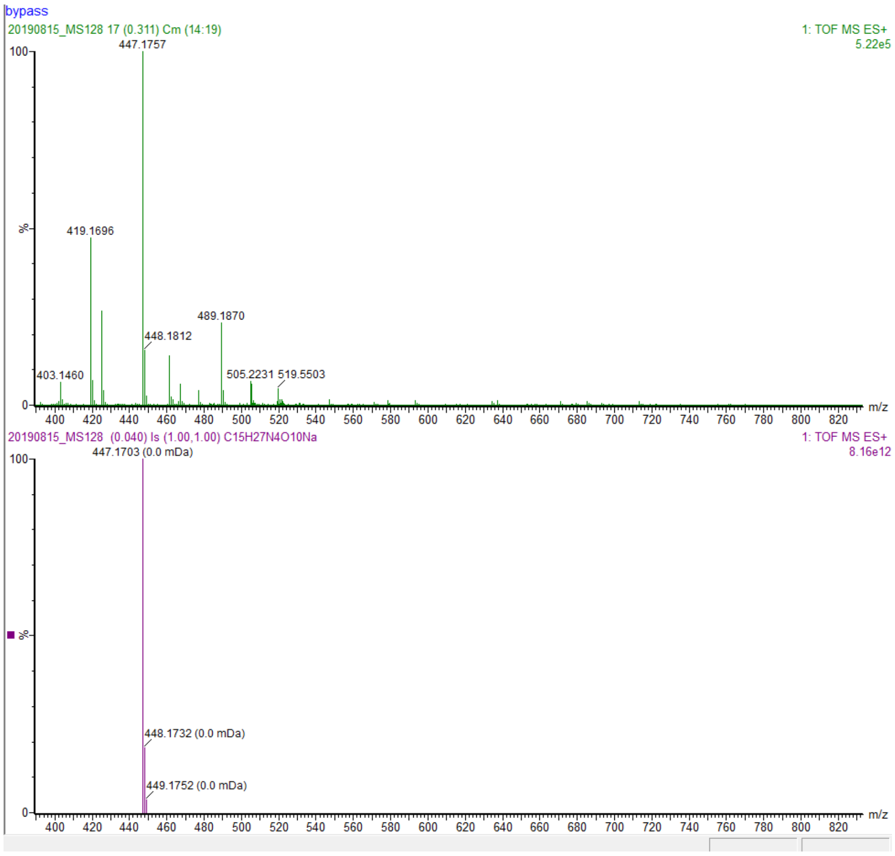

Figure S12. Mass spectrum of compound **6.**

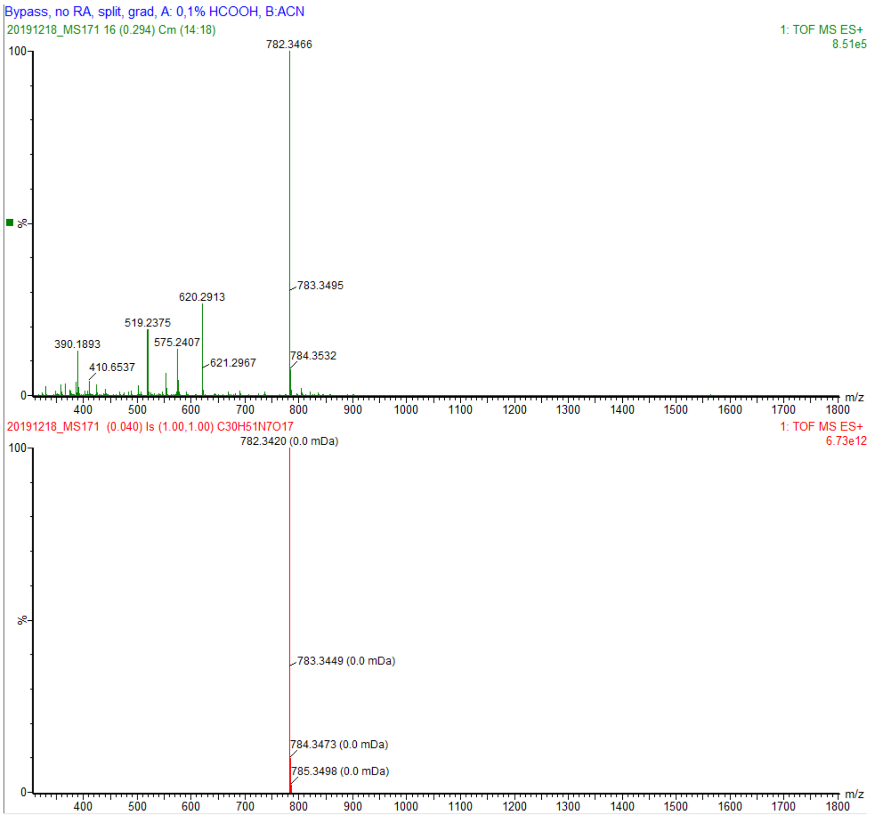

Figure S13. Mass spectrum of compound **8.**

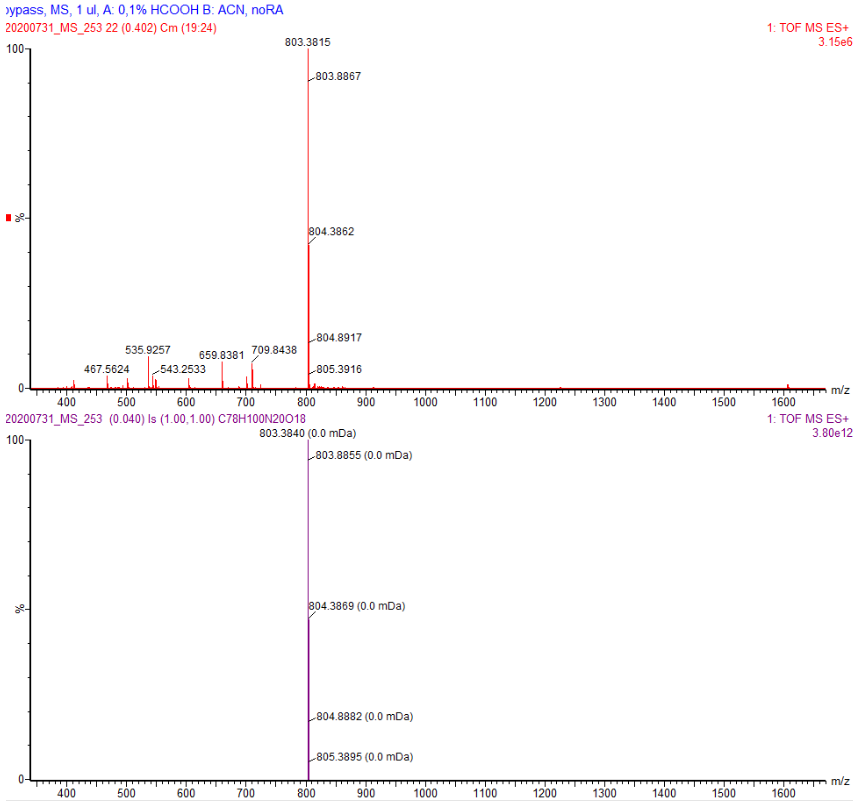

Figure S14. Mass spectrum of compound **11.**

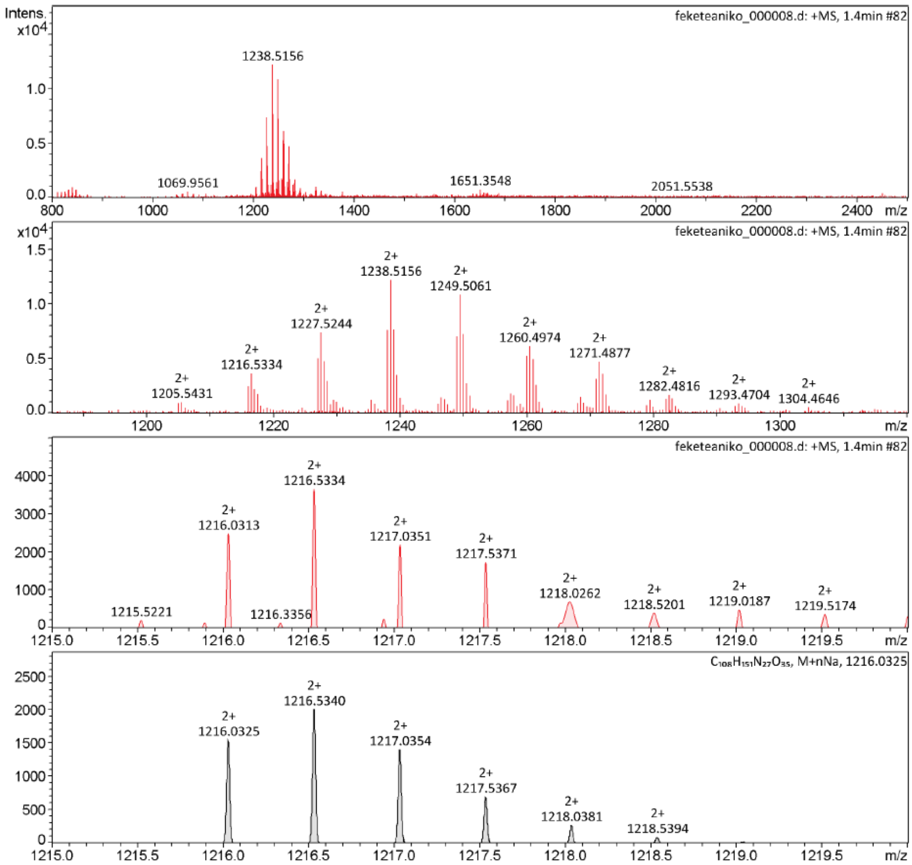

Figure S15. Mass spectrum of compound **12.**
